# Comparison of Overnight Pooled and Standard Sputum Collection Method for Patients with Suspected Pulmonary Tuberculosis in Northern Tanzania

**DOI:** 10.1155/2012/128057

**Published:** 2012-01-19

**Authors:** Stellah G. Mpagama, Charles Mtabho, Solomon Mwaigwisya, Liberate J. Mleoh, I Marion Sumari-de Boer, Scott K. Heysell, Eric R. Houpt, Gibson S. Kibiki

**Affiliations:** ^1^Kibong'oto National Tuberculosis Referral Hospital, P.O. Box 12, Kilimanjaro, Tanzania; ^2^Kilimanjaro Clinical Research Institute and Kilimanjaro Christian Medical College, Kilimanjaro Moshi, Tanzania; ^3^Division of Infectious Diseases and International Health, University of Virginia, Charlotteville, VA 22908, USA

## Abstract

In Tanzania sputum culture for tuberculosis (TB) is resource intensive and available only at zonal facilities. In this study overnight pooled sputum collection technique was compared with standard spot morning collection among pulmonary TB suspects at Kibong'oto National TB Hospital in Tanzania. A spot sputum specimen performed at enrollment, an overnight pooled sputum, and single morning specimen were collected from 50 subjects and analyzed for quality, quantity, and time to detection in Bactec MGIT system. Forty-six (92%) subjects' overnight pooled specimens had a volume ≥5 mls compared to 37 (37%) for the combination of spot and single morning specimens (*P* < 0.001). Median time to detection was 96 hours (IQR 87–131) for the overnight pooled specimens compared to 110.5 hours (IQR is 137 right 137–180) for the combination of both spot and single morning specimens (*P* = 0.001). In our setting of limited TB culture capacity, we recommend a single pooled sputum to maximize yield and speed time to diagnosis.

## 1. Background

Tuberculosis (TB) and HIV are among the global leaders in infectious disease mortality [1]. Sub-Saharan Africa has one of the highest burdens of TB and HIV coinfection [2]. Prompt diagnosis of TB is critical to improve outcome, but diagnosis of TB is challenging in HIV-infected patients and especially in resource-limited settings [3]. HIV-infected patients have a higher rate of extrapulmonary TB, atypical chest radiographs and fewer pulmonary cavities [4–6]. As a consequence, HIV patients may be more likely to have a negative or paucibacillary sputum smear microscopy [7]. Subjects with paucibacillary specimens may be prone to being delayed in clinical diagnosis, either because acid-fast bacilli are not observed by microscopy or time to detection *Mycobacterium tuberculosis* (MTB) culture is prolonged [8]. Both poor quality and low quantity of sputum have a significant impact on TB detection rate [9, 10]. In settings reliant on smear microscopy as the only means of TB diagnosis, this may impact negatively the time to initiation of TB treatment. Ideal specimens should contain 5 mL or more of sputum without saliva. A previous study found that the quality and quantity of sputum were improved by pooling three versus a single “spot” collection [11]. However, collection on multiple days may unnecessarily burden health facilities and may be prone to contamination. In contrast, a single overnight pooled technique whereby a patient is given a sealable container in which to collect all expectorated sputum over the course of the night has been used in TB treatment trials and in assessment of early bactericidal activity of new TB medications [12, 13]. However it has not been examined in routine clinical practice.

At Kibong'oto National TB Hospital in Kilimanjaro, Tanzania, HIV co infection among TB suspects is common and it has been observed that the minority of patients have cavitary chest radiographs. Furthermore it is a standard practice that each TB suspect is given a sealable mug to collect all sputum as an infection control measure. Thus, among a population at risk for lower bacillary yield, we sought to compare overnight pooled sputum in the sealable mugs to the standard spot technique for sputum quality, quantity, and time to MTB detection by culture in the Bactec MGIT system, a method which has correlated well with conventional colony counts [14, 15].

## 2. Methods

**Figure 1:**
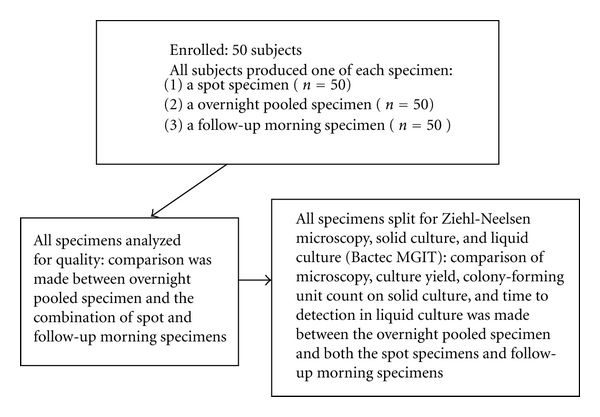
Study diagram of enrollment and specimen analysis.

Subjects suspected of pulmonary TB at Kibong'oto National TB Hospital were recruited for enrollment. Subjects were eligible if they were 18 years and above and presenting with two weeks or more of cough. Each patient was asked to collect three sputum samples in calibrated wide mouthed container: (1) a spot specimen at the time of enrollment, (2) an overnight pooled specimen, and (3) a spot specimen on the morning after enrollment (Figure 1). Subjects were excluded if initiated on any antituberculosis medication prior to sample collection. Sputum samples were sent to Kilimanjaro Clinical Research Institute (KCRI) laboratory for processing. The specimens were assessed for volume collected, presence of blood, and color. Samples were analyzed for quality based on the following criteria: (1) volume equal to or more than 5 mls, (2) sputum color was white, yellow, green, or red, (3) presence of blood, and (4) thick consistency. Each sputum specimen was decontaminated with 4% NaOH and centrifuged at 1500 g for 10 minutes. The sediment was split equally for use in direct smear microscopy by Ziehl-Neelsen staining, MTB culture on solid agar with Lowenstein-Jensen slant and Middlebrook 7H11 media, and in liquid media using Bactec MGIT 960 machine (Becton Dickinson, USA). Positive culture growth was confirmed by secondary Ziehl-Neelsen stain. Colony-forming unit (CFU) count was performed on the Middlebrook 7H11 media.

Data were analyzed by Stata Version 11 statistical software (StataCorp, U.S.A). Values were presented as means with standard deviation (SD) or median with interquartile range (IQR) for data that was not normally distributed. Chi-square was used for dichotomous variables and Spearman's correlation coefficient for continuous variables to investigate the association with microscopy and culture yield. All tests were two sided with *P* values ≤ 0.05 regarded as statistically significant.

All subjects provided written informed consent. Ethical approval for the study was given by the IRB of the Kilimanjaro Christian Medical Center, Tumaini University and the National Ethical Review Board.

## 3. Results

Fifty pulmonary TB suspects were enrolled for the study, each produced three specimens, hence 150 samples were used for analysis. The median age of patients was 38.5 years (IQR 30–45) (Table 1). Thirty-six (72%) were male and 8 (16%) were HIV-1-infected patients. Among the HIV-infected subjects, the median CD4 count was 71 cells/*μ*L (IQR 30.5–158). The median CD4 count for HIV-seronegative patients was 540 cell/*μ*L (IQR 409–614). Twenty-three (46%) had cavitary disease (Table 1).

Overnight pooled sputum yielded a specimen of ≥5 mL in 46 (92%) of subjects compared to 37 (37%) for the combination of spot and single morning specimens (*P* < 0.001). Good quality of sputum specimen by color was observed in 47 (94%) of the patients for overnight pooled sputum—while combination of spot and single morning specimens was 92 (92%) (*P* = 0.79). Thick consistency was observed in 41 (82%) of patients for overnight pooled compared to combination of spot and single morning 66 (66%) (*P* = 0.12), and presence of blood was observed in 6 (12%) and 11 (11%), respectively (*P* > 0.99) (Table 2).

**Figure 2:**
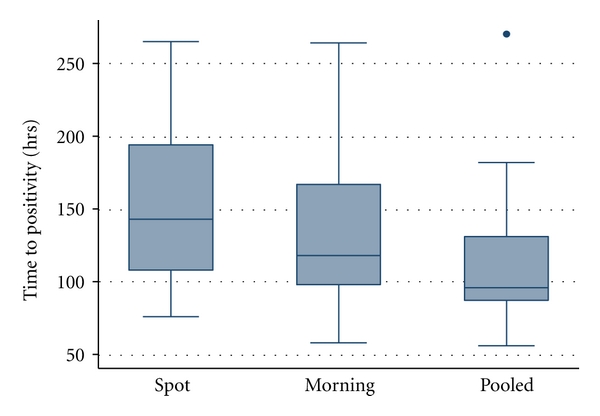
Median time-to-culture positivity among different collection method. *P < 0.005 comparing pooled to morning or spot.

Overall 100 (67%) specimens were positive by smear microscopy. Thirty-four (68%) spot specimens were smear positive, compared to 31 (62%) single morning specimens, and 35 (70%) for overnight pooled (*P* = 0.28). Eighty-one (54%) culture-positive specimens were available for comparison of time to detection in the MGIT system from 27 subjects; 49 (33%) specimens were negative and bacterial contamination precluded analysis in 20 (13%). The MGIT contamination rate did not differ from that of Lowenstein-Jensen, found in 21 (14%) of all specimens. Furthermore, among MGIT specimens contamination did not vary by method of collection: spot (5.3%), single morning (3.3%) and overnight pooled (4.7%) (*P* > 0.99). However, the median time to detection was 96 hours (IQR 87–131) for the overnight pooled specimens which was significantly faster compared to 118 hours (IQR 98–167) for single morning specimen and 143 hours (IQR 108–194) for the spot specimens (*P* < 0.005) (Figure 2).

Only a limited number of pooled specimens were TB-culture positive on Middlebrook 7H11 to allow colony forming unit (CFU) count determination. The median CFU count among subjects with cavities on chest radiograph was 4.92 log/mL (IQR 4.3–6.28) compared to 3.95 log/mL (IQR 3.61–4.84) in patients without cavities (*P* = 0.12). Among the 8 HIV-infected subjects, only 2 (25%) had overnight pooled specimens available for CFU count. The median CFU count among HIV-infected subjects was 4.34 log/mL (IQR 3.9–4.72) compared to 4.84 log/mL (IQR 4.30–5.86) for the HIV-uninfected subjects (*P* = 0.43). There was no correlation between CD4 counts with CFU (Spearman's rho = −0.36) (*P* = 0.16).

## 4. Discussion

The major finding of this study was that in routine practice an overnight pooled sputum collection significantly improved the time to detection in the MGIT system among culture-positive samples. This is an important finding amidst the global scale-up of MTB culture.

Faster time to diagnosis in a TB suspect can speed drug susceptibility testing, hasten proper treatment, and potentially decrease transmission to the community and within the hospital. In settings with increasing rates of multidrug-resistant (MDR) TB, faster drug susceptibility results have been postulated to be one of the most important means of improving MDR TB outcome [16].

Sputum collection is the first and most critical step in laboratory diagnosis of pulmonary TB. In resource-limited settings such as ours, multiple specimen collection has been proposed to increase yield for rapid molecular probe diagnostics [17, 18]; however it can be overwhelming to the laboratory. Both microscopy and culture are time consuming and culture is costly. In Tanzania culture is recommended for smear-negative cases, for treatment failures and for monitoring MDR-TB patients. Currently there are few laboratories capable of culture and specimens must be sent long distances. In this setting, maximizing yield by sending the optimal specimen will conserve resources. We would strongly advocate sending pooled specimens in our context.

Our findings of increased culture yield with volumes of a minimum of 5 mls of adequate quality are consistent with previous studies that examined yield of microscopy [19, 20]. However, our microscopy yield did not approach the remarkable >95% rate of smear positivity reported by others when a 24-hour collection technique was used for diagnosis of selected subjects with pulmonary TB [21]. This is potentially contributed by the fact that we selected for pulmonary TB suspects and not confirmed cases. With an eye towards molecular diagnostics in the future, given the drop in sensitivity of the rapid molecular diagnostics in smear-negative sputum samples [17], we would expect that overnight pooled sputum collection would be equal to or improve upon molecular diagnostic yield compared with a spot specimen.

Our study has several limitations. For the CFU analysis on the overnight pooled specimens, fungal overgrowth limited examination of nine different plates. The contamination affected subjects with and without cavities so it is unlikely to have biased this finding; yet overall our total number of patients enrolled was small and may not have included enough HIV-infected patients to appreciate differences in this subgroup with regard to culture yield, CFU analysis, and time to detection. Bacterial contamination also limited evaluation of all samples in the MGIT, but the contamination was observed in all specimen collection techniques and has been reported in other evaluations of liquid culture in MGIT [22]. Furthermore, the culture yield for all techniques may have been diminished by splitting specimens for both solid and liquid culture.

In conclusion, we found that overnight pooled sputum collection improved the quantity of sputum without alteration in quality and led to faster time to detection. These findings can ultimately impact the decisions both of treatment and infection control. Overnight pooled sputum should be considered as an alternative to standard spot testing in resource-limited settings.

## Figures and Tables

**Table 1:** Demographic and clinical characteristics.

Variables (*N* = 50)	Measure
Sex	
Male	36 (72%)
Age (years) median (IQR)	38.5 (30–46)
Weight (kg) median (IQR)	51 (47–58)
HIV status	
Positive	8 (16%)
CD4 count (cells/*μ*L) by HIV status in median (IQR)	
HIV positive	71 (30.5–158)
HIV negative	540 (409–614)
Chest X-ray findings	
Cavities	23 (46%)

**Table 2:** Comparison of overnight pooled and spot method of collection on quality and quantity of sputum.

Measure	Combination of spot and single morning specimen	Overnight pooled	*P* value
Quantity (≥5 mls)	37/100 (37%)	46/50 (92%)	*P* < 0.001
Color	92/100 (92%)	47/50 (94%)	*P* = 0.79
Thick consistency	66/100 (66%)	41/50 (82%)	*P* = 0.12
Presence of blood	11/100 (11%)	6/50 (12%)	*P* > 0.99

*P values are similar if pooled is compared with spot or single morning specimen, thus we combined spot and single for this table.
